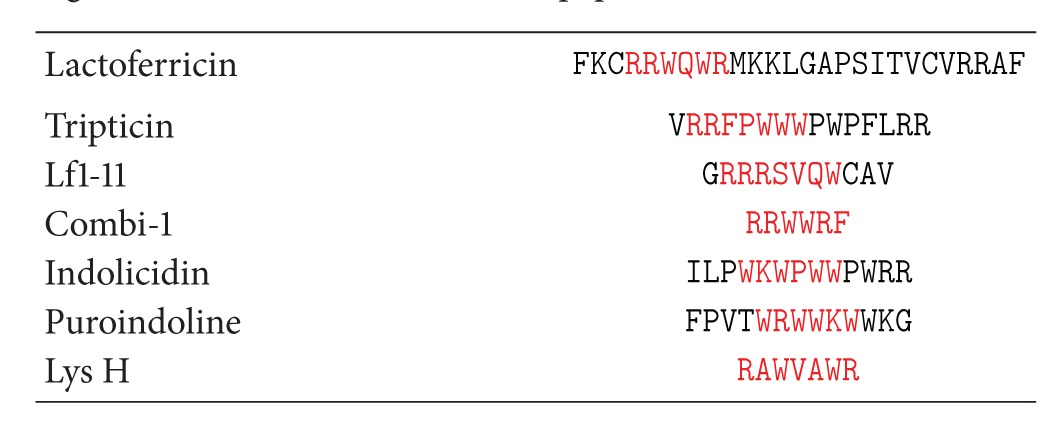# Antimicrobial Lactoferrin Peptides: The Hidden Players in the Protective Function of a Multifunctional Protein

**DOI:** 10.1155/2013/390230

**Published:** 2013-02-13

**Authors:** Mau Sinha, Sanket Kaushik, Punit Kaur, Sujata Sharma, Tej P. Singh

**Affiliations:** Department of Biophysics, All India Institute of Medical Sciences, Ansari Nagar, New Delhi 110029, India

## Abstract

Lactoferrin is a multifunctional, iron-binding glycoprotein which displays a wide array of modes of action to execute its primary antimicrobial function. It contains various antimicrobial peptides which are released upon its hydrolysis by proteases. These peptides display a similarity with the antimicrobial cationic peptides found in nature. In the current scenario of increasing resistance to antibiotics, there is a need for the discovery of novel antimicrobial drugs. In this context, the structural and functional perspectives on some of the antimicrobial peptides found in N-lobe of lactoferrin have been reviewed. This paper provides the comparison of lactoferrin peptides with other antimicrobial peptides found in nature as well as interspecies comparison of the structural properties of these peptides within the native lactoferrin.

## 1. Introduction

 The innate immune system or the nonspecific immune system is the first and the oldest line of defense in organisms [1, 2]. It was the most dominant form of immunity before the evolution of the more sophisticated adaptive immunity. It is comprised of various mechanisms which are responsible for rapid defense of the host organism against invasion by other factors in a nonspecific manner. The innate immune system differs from the adaptive immune system in a way that while it is able to defend the body against pathogens, it is not able to impart long-lasting immunity to the host, unlike the latter [3]. Despite the evolution of the more complex and specific adaptive immunity, innate immunity still continues to function as the primary line of defense for most organisms [4]. The antimicrobial action in the innate immunity is mediated by various antimicrobial proteins and peptides, which have been evolutionary conserved. Antimicrobial peptides are small peptides which demonstrate broad-spectrum antibiotic activity against various gram-positive and gram-negative bacteria, fungi, protozoa, and viruses [5–8]. While the most common mechanism of action deployed by these peptides is perturbation of microbial cell membrane [9–11], there are other mechanisms which are also prevalent [12–16]. Due to increasing resistance to antibiotics, there is an urgent requirement of novel antimicrobial drugs [17–19]. Use of antimicrobial peptides is one of the promising approaches which may lead to potential antimicrobial drugs [16, 20–24]. It has been observed that peptides which are predominantly cationic and hydrophobic in nature show potent antimicrobial activity [5, 25–29]. Many of these peptides including indolicidin from bovine neutrophiles [30], tripticin from porcine neutrophil granules [31], puroindoline from wheat seeds [32], combi-1, a synthesized antimicrobial peptide [33], and Lys H and Lys C from lysozyme [34] have been extensively studied. Although these peptides adopt various conformations the alpha-helical conformation with polar and nonpolar groups on opposite sides of the helix tends to be the most abundant [35–37]. The antimicrobial property of these amphipathic alpha-helical peptides increases sequentially with increase in net charge [38].

 Lactoferrin is an iron-binding glycoprotein which is found in most of the exocrine secretions such as milk, tears, nasal secretions, saliva, urine, uterine secretions, and amniotic fluids [39–41] as well as in secondary granules of neutrophils [42]. It exerts a wide antimicrobial activity against a number of bacterial, viral, and fungal pathogens in vitro [43–48]. Lactoferrin exerts its antimicrobial action not just in the form of the intact molecule but the monoferric lobes and active peptides of lactoferrin also have a role in the host defense against microbial disease [49–52]. Lactoferrin is a rich source of cationic and hydrophobic antimicrobial peptides, which may be used against microbes [53, 54]. These antibacterial peptides which are a part of the polypeptide chain of lactoferrin and are released upon the proteolysis of this molecule by various proteolytic enzymes can be developed into clinically useful lead molecules for antimicrobial therapeutics [55, 56]. 

Although it has been shown that native lactoferrin exerts its antimicrobial action through sequestration of iron [57–59], it is still unclear how the antimicrobial lactoferrin peptides act against the microbes. Though a number of studies have implicated these peptides in the binding to the outer membrane proteins of various bacteria or binding to microbial proteases [60–62], the structure-function interrelationships of these peptides have not yet been established. 

**Figure 1:**
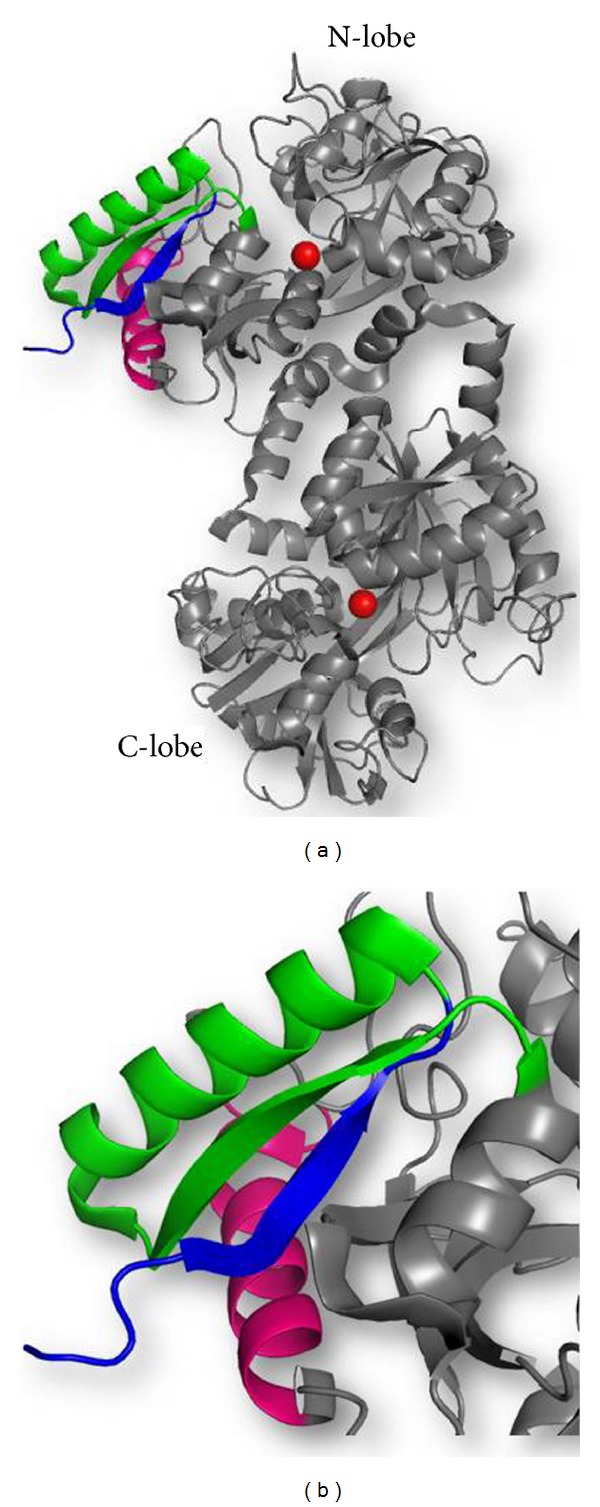
(a) Overall structure of lactoferrin showing positions of LF1-11 (blue), lactoferrampin (pink), and lactoferricin (green) peptides in the N-terminal lobe. (b) The zoomed structure showing the position of peptides in detail.

**Figure 2:**
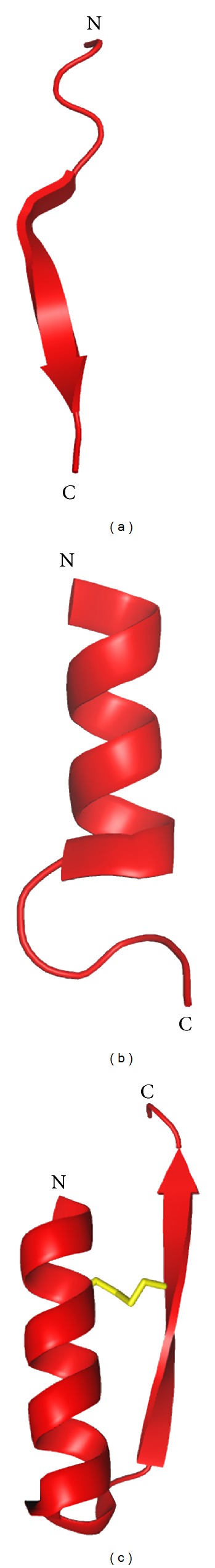
The structural comparison of peptides (a) LF1-11, (b) lactoferrampin, and (c) lactoferricin in the native structure of human lactoferrin (PDB : 1LFG).

A number of functional peptides are produced from lactoferrin by the action of proteolytic enzymes. It is expected that these enzymes are present in the gastrointestinal tract as well as the site of microbial infection, and hence, they may contribute in the natural function of lactoferrin in the human body. This paper reports the comparison of these peptides with other antimicrobial peptides found in nature as well as cross-species comparison of the sequences of these antimicrobial peptides from the native sequences of lactoferrin with the intent to draw evolutionary inferences of their function. Although many antimicrobial peptides from lactoferrin have been isolated and characterized, only three of them have been studied in detail. These are LF1-11, lactoferrampin, and lactoferricin. The sequences of these peptides indicate that these peptides belong to the N-terminal half of lactoferrin (Figure 1). Hydrophobicity, cationicity, and helical conformation of these antimicrobial peptides are the important characteristics that determine their antimicrobial potency [9, 63, 64]. All these peptides have high pI values (>9) and is expected to interact with negatively charged elements. All three have different sequences, structural elements, and modes of action (Table 1). An attempt to analyze and decipher the structural and functional characteristics of three peptides is made in this paper. Their overall structural comparison as observed in intact lactoferrin is depicted in Figure 2.

## 2. LF1-11

LF1-11, as its name suggests, is the N-terminal peptide of lactoferrin, comprised of the first eleven residues of the molecule. This peptide has been shown to be highly effective against five multidrug-resistant *Acinetobacter baumannii* strains [65] and methicillin-resistant *Staphylococcus aureus* [66] and various *Candida* species [67, 68]. The potent antimicrobial effect of LF1-11 was attributed to the first two arginines at the N-terminus of human lactoferrin [69]. This conclusion was based on the fact that when the second or third arginines were replaced by alanine, the candidacidal activity of the LF1-11 was observed. Additionally, while LF1-11, LF2-11, and LF3-11 showed comparable candidacidal activities, the same was found compromised in the case of LF4-11.

The importance of the three arginines (R2–R4) for the potent antimicrobial activity of this peptide was established when synthetic peptides lacking the first three N-terminal residues were found to be less effective [70] in the killing of bacteria. Also, mutant lactoferrin lacking the first five N-terminal residues displayed decreased binding to bacterial lipopolysaccharide [71]. 

In yet another study, Stallmann et al. studied the efficacy of local prophylactic treatment with human LF1-11 in a rabbit model of femur infection and observed that hLF1-11 effectively reduced the development of osteomyelitis in a rabbit model [72]. 

It was speculated that the mechanism of antimicrobial action of LF1-11 is mitochondrial damage, with the extracellular ATP being essential but not sufficient for LF1-11 to exert its candidacidal activity [69]. In later studies, it was found that uptake of calcium by mitochondria is vital for killing of *Candida albicans* by the LF1-11 [73]. 

In another study, it was found that LF1-11 is responsible for directing the GM-CSF-driven monocyte differentiation toward macrophages that produces both pro- and anti-inflammatory cytokines. It was speculated that the peptide could be used as agent to empower the innate immune response of the host for infections. These results demonstrated the importance of the further development of LF1-11 as a promising drug against microbial infections in patients who may have compromised immune systems [74].

The cellular target for the immunomodulatory activity of LF1-11 was found to be myeloperoxidase, to which LF1-11 binds and inhibits after entering the monocytes. A molecular modeling study by the same group demonstrated that LF1-11 bound at the active site of the enzyme. The importance of the first two arginines and the cysteine at the tenth position was further substantiated by the fact that peptides which did not possess these necessary residues were not as effective in binding with myeloperoxidase [75]. 

The sequence comparison of LF1-11 among the six species (Table 2) shows that unlike human LF1-11, which contains three arginines in the positions 2–5 (R2–R4), the peptide from other species contains only one arginine (R3). Yet, it is noteworthy that the R4 has been replaced by lysine, which is also a basic residue in all the other species, thereby maintaining the highly cationic nature of the peptide throughout the species. Also, in all the cases except human, arginine occurs at the seventh position also. The most significant change is seen in the second residue which is proline in all cases except in human and camel. Notably, the hydrophobic residues, V6, and W8 are conserved in all the cases.

## 3. Lactoferrampin 

Lactoferrampin, comprised of residues 268–284 in the N1 domain of lactoferrin, has been identified as an antimicrobial peptide and plays a key role in membrane-mediated activities of lactoferrin [76, 77]. It exhibits broad antimicrobial action against several gram-positive and gram-negative bacteria, notably, *Bacillus subtilis*, *Escherichia coli*, *Pseudomonas aeruginosa*, and *Staphylococcus aureus*, as well as candidacidal activity [76]. 

The antimicrobial action of this peptide was also found to be more potent than the native lactoferrin. This peptide was found to be located in close proximity to lactoferricin. The structure of lactoferrampin revealed an amphipathic alpha-helix which begins with the N-terminus and ends at the 11th residue, followed by a C-terminus tail [78]. 

It is reported that the cleavage of this peptide at both the termini resulted in considerable decrease of the candidacidal activity. The C-terminal residues of lactoferrampin are most critical for its antimicrobial action, possibly because the C-terminus consists of several residues with positive charges which are clustered together. But truncation of C-terminal side did not alter the ability of this peptide to adopt helical conformations. Also, substitution of the basic residues at the C-terminus led to decrease in potency of this peptide [77, 79]. The N-terminal residues, truncated up to the sequence 270–284, are essential for maintaining the structure of this peptide in a helical conformation [77].

The helical conformation of this peptide was found to be critical for the potency against gram-positive bacteria as established when the bactericidal activities of two lactoferrampin peptides, lactoferrampin 265–284 and lactoferrampin 268–284, were compared [80]. Lactoferrampin 265–284, which consists of additional three residues, Asp-Leu-Ile, showed a broader specificity since the Asp-Leu-Ile sequence increases the tendency of this peptide to assume an alpha-helical conformation. Both the peptides possessed bactericidal activity against certain species of gram-positive and gram-negative bacteria. Compared to lactoferrampin 268–284, higher concentrations of lactoferrampin 265–284 were required to kill the gram-negative bacteria, *E. coli* and *P. aeruginosa*. The killing activity expressed as LC_50_ value (the concentration that produced 50% reduction in viable counts of the microorganisms) was found to be about 5.8 *μ*mol/L for lactoferrampin 268–284 which is about 4 times higher than lactoferrampin 265–284 [80].

The mode of action of this peptide on bacteria is by bacterial membrane binding and membrane disruption. It is established that lactoferrampin is internalized within few minutes with the bacterial membrane permeabilization followed by cellular damage [81, 82].

Distinct vesicle-like structures by the lactoferrampin peptide were also observed by freeze-fracture transmission electron microscopy in the membrane of *C. albicans* [81]. It is speculated that this peptide exerted detergent-like activity, disturbing the hydrophobic interphase of the lipid bilayer. 

Several studies have revealed that the determinants for antimicrobial action are the orientation and structure of bovine lactoferrampin in bacterial membranes [78, 83–85]. The solution structure of bovine lactoferrampin suggests that it adopts an amphipathic alpha-helical conformation across the first 11 residues of the peptide but remains comparatively random at the C-terminus [78, 85]. The interaction between the N-terminal tryptophan residue and model membranes of varying composition was evaluated suggesting that W1 is inserted into the membrane at the lipid/water interface [78]. Along with this, the orientation of the phenyl side chain of F11 found to be in same direction as the indole ring of W1 also suggested that the amphipathic N-terminal helix anchors the peptide to membrane with these two residues that facilitates peptide folding [78, 86]. The same group has suggested that the hydrophobic patch in between the two residues as well as Leu, Ile, and Ala side chains are responsible for interaction between the peptide and the hydrophobic core of a phospholipid bilayer [83]. In addition, the helix capping residues Asp-Leu-Ile in the N-terminus of the peptide has been found to mediate the depth of membrane insertion by enhancing the affinity for negatively charged vesicles [84]. Bovine lactoferrampin had been shown to have greater affinity to acidic phospholipids than that to neutral phospholipids [85]. Haney et al. have speculated a two-step model of antimicrobial action by this peptide where the C-terminus positive charge cluster helps in the primary attraction of lactoferrampin to the membrane followed by the helix formation at the N-terminus that interacts to the surface of the bacterial lipid bilayer [78].

The sequence comparison of lactoferrampin from six different species shows uniform preponderance of cationic amino acid residues among hydrophobic residues (Table 3). The hydrophobic domain contains W1 in all the species that is involved in membrane insertion [87]. Bovine lactoferrampin 268–284 has a net positive charge of 5+ at neutral pH with hydrophobic domain. The hydrophobic moment (*μ*) of the peptide which is a measure of lipophilicity was found to be 5.42. In contrast, the human lactoferrampin has a net charge of 2+ resulting in reduced antimicrobial activity [76]. However, by increasing the net positive charge near the C-terminal end of human lactoferrampin, a significant increase in its antibacterial and candidacidal activity was obtained [83]. The basic amino acid residues crucial for the antimicrobial action were found to be conserved among all the six species. 

## 4. Lactoferricin

Lactoferricin is a multifunctional, 25-residue peptide that is generated upon cleavage of native lactoferrin by pepsin and represents amino acid residues number 17–41 in lactoferrin. The lactoferricin peptide is different from the other peptides described so far as it contains a disulfide bond between residues Cys 20 and Cys 37 in human lactoferrin and Cys 19 and Cys 36 in bovine lactoferrin. The peptide has an abundance of basic amino acids like lysine and arginine as well as hydrophobic residues like tryptophan and phenylalanine.

The first report on lactoferricin in 1992 described this peptide to be more potent as an antibacterial agent in comparison with the intact lactoferrin and it was demonstrated to cause a rapid loss of colony-forming capacity in most of its targets. However, some strains like *Pseudomonas fluorescens*, *Enterococcus faecalis*, and *Bifidobacterium bifidum* strains were found to be resistant to lactoferricin [88]. 

The antibacterial activity of this peptide was attributed to its action of releasing lipopolysaccharide from bacterial strains and, hence, disruption of cytoplasmic membrane permeability after cell binding [89–91]. Apart from having a broad antibacterial spectrum, lactoferricin was found to be highly potent against *Candida albicans* [89, 92, 93]. Recently, it has also been shown to have antiviral [94, 95] and antiprotozoal activities [96]. It also displayed other activities like inhibition of tumor metastasis [97] and induction of apoptosis in human leukemic cells [98]. 

The mechanism of action of lactoferricin was attributed to 11-amino-acid amphipathic alpha-helical region which is positioned on the outer surface of the N-lobe of lactoferrin. The proline at the 26th position (P26) was found to be essential for the antibacterial activity, and it was speculated to be responsible for disruption of the helical region, and hence the helicity of the peptide was predicted to be an essential aspect of the antibacterial action of this peptide [99]. Lactoferricin was found to be produced in the human stomach, indicating that this peptide is definitely generated in vivo for host defense [100]. 

The comparison of the antimicrobial activities of lactoferricin from human, bovine, murine, and caprine showed that bovine lactoferricin was the most potent [101]. The minimal inhibitory concentration (MIC) of lactoferricin B differs according to their source [102]. A comparison of the MIC values of lactoferricin shows that bovine lactoferricin is the most potent. The MIC of bovine lactoferricin against certain *E. coli* strains has been found to be around 30 *μ*g/mL while that derived from human is more than 100 *μ*g/mL. The efficiency of antibacterial activity of bovine lactoferricin is due to the presence of high amount of net positive charge (+8) and hydrophobic residues (primarily W6, W8, and M10) [51, 88]. The action of this peptide is also dependent on the pH [89, 103]. 

It was shown that only six central residues (4–9) among the twenty five residues of the peptide are required for its antimicrobial activity [104]. It may be noted that the tetrapeptide KRDS is present only in human lactoferrin while it shows variations in the sequence of others. It has been reported that KRDS inhibits platelet aggregation [105, 106]. 

The mode of action of lactoferrin peptides is best studied in bovine lactoferricin. The bovine lactoferricin has been demonstrated to interact with the negatively charged elements in the membrane of susceptible bacteria and disrupt the cell membrane. A synthetic peptide derived from human lactoferricin has been found to be effective in depolarizing the bacterial cytoplasmic membrane with a loss of pH gradient [107]. 

The permeabilizing effect of bovine lactoferricin causes membrane disruption resulting in inhibition of macromolecular biosynthesis and ultimately cell death [108]. The mode of action is however different in gram-positive and gram-negative bacteria. In gram-negative bacteria antimicrobial peptides act on lipopolysaccharides and in gram-positive bacteria they act on lipoteichoic and teichoic acids. In addition to antimicrobial properties, lactoferricin derived from human and bovine origin has also been found to be effective in inhibiting the classical complement pathway. This implicates a role of these peptides in suppression of inflammatory effects caused by bacteria [109].

The sequence analysis of the lactoferricin from various species indicates that unlike bovine lactoferricin there is only one tryptophan at position 6 (Table 4). A further exception to this is equine which has no tryptophan residue in either of the positions. This shows that the two tryptophans in lactoferricin are important for its optimal activity against microbes [110]. 

The solution structures of lactoferricin have been determined from bovine [111] and human [112] sources. The bovine lactoferricin adopted a distorted antiparallel beta sheet, in complete contrast with its conformation in the intact lactoferrin, as observed in the structures obtained by X-ray crystallography. However, the solution structure of human lactoferricin was closer to its structure in native lactoferrin since the amphipathic helix was preserved from Gln14 to Lys29. However, the beta-sheet character was not observed in the solution structure of human lactoferricin either.

## 5. Lipopolysaccharide Neutralization Activity of Lactoferrin-Derived Peptides

Lipopolysaccharide (LPS), the outer membrane component of gram-negative bacteria, is one of the major causes of endotoxin-induced production of inflammatory cytokines [113] and septic shock [114]. Lactoferrin has been shown to neutralize the effect of LPS-induced toxicity by binding to LPS [115, 116]. The cationic peptide derived from lactoferrin which is responsible for this interaction and release of LPS is first identified to be lactoferricin [90]. The residues from 28 to 34 of lactoferrin corresponding to the region in human lactoferricin have been identified to have a high affinity for binding to LPS [117]. Soluble LPS can interact with bovine lactoferricin. The initial binding of the peptide with *E. coli* has been found to be due to interaction with bacterial LPS [118]. Further studies have shown that bovine lactoferricin can arrest the LPS-induced cytokine release by suppressing the IL-6 response in human monocytic cells stimulated by LPS [119]. A synthetic peptide corresponding to the antibacterial region of human lactoferricin was also found to facilitate depolarization of the bacterial cytoplasmic membrane, loss of the pH gradient, and a bactericidal effect in *E. coli* [60]. Modelling studies using synthetic peptides derived from human and bovine lactoferricin have shown that these cationic peptides with their positively charged residues first interact with LPS carrying negative charges. This is followed by hydrophobic interactions between the tryptophan residues of the peptides and the lipid A molecule of LPS to promote structural disorganization [120]. Similarly, a synthetic peptide corresponding to 11 residues of human lactoferricin near its N-terminus has been found to bind to LPS and neutralize the LPS-induced adverse effects in vitro and in monocytes [121, 122]. In yet another study using NMR, it has been observed that this peptide folds into a “T-shaped” conformation formed by its hydrophobic core and the two clusters of hydrophilic residues of the peptide targets the two phosphate moieties of lipid A in LPS [123].

## 6. Comparison of Lactoferrin Antimicrobial Peptides with Other Antimicrobial Peptides Found in Nature 

The antimicrobial peptides found in nature are classified into four groups according to a combination of their sequence homologies, functional similarities, and common three-dimensional structures [124]. 

The four groups include Group 1, which consists of linear, cationic, and amphipathic-helical peptides, for example, cecropins, magainins, bombinins, and temporins; Group 2, which consists of *β*-strands connected by intramolecular disulfide bridges, for example, human *β*-defensin-2, tachyplesins, and protegrins; Group 3, which consists of linear peptides with an extended structure, characterized by overrepresentation of one or more amino acids, for example, tritrpticin and indolicidin; and Group 4, which consists of peptides containing a looped structure, for example, bactenecin, brevinins, and esculentin. 

In the light of the above classification, human LF1-11 (GRRRSVQWCAV) consists of a highly variable loop region and a short *β*-strand and is arginine rich, and hence can be classified in Group 4. However, the same cannot be said about LF1-11 from other species, since their conformation may be similar to the human LF1-11 in the structure, but they are not rich in arginines. The arginine-rich fragment of this peptide is similar to other cationic arginine-rich peptides found in nature which have cell-penetrating activity and hence can traverse the plasma membrane of eukaryotic cells [125]. A significant example of arginine-rich peptide that has cell-penetrating property is arginine-rich HIV Tat peptide (GRKKRRQRRRPPQ) [126].

**Figure 3:**
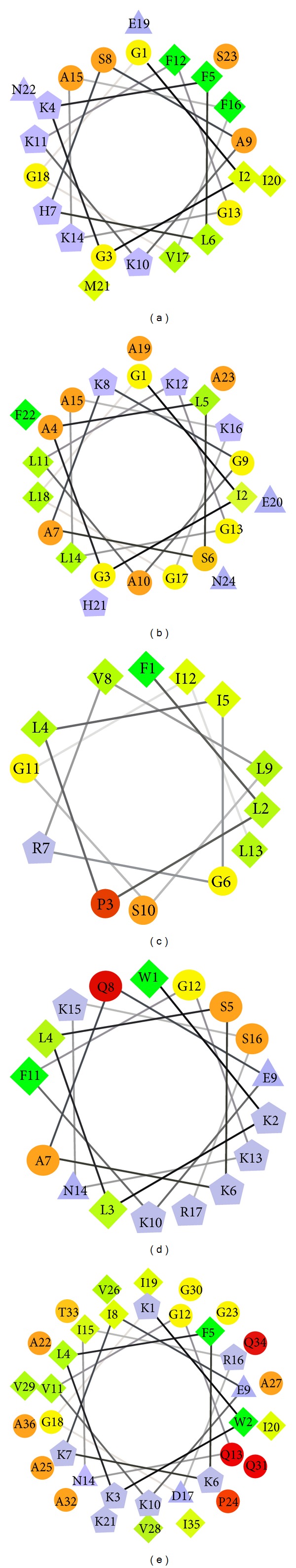
Helical wheel representation of (a) magainin 2, (b) bombinin, (c) temporin, (d) lactoferrampin, and (e) cecropin A. The hydrophilic residues are shown as circles, hydrophobic residues as diamonds, potentially negatively charged as triangles, and potentially positively charged as pentagons. Hydrophobicity is color coded as well: the most hydrophobic residue is green, and the amount of green is decreasing proportionally to low hydrophobicity, coded as yellow. Hydrophilic residues are coded red with pure red being the uncharged residues, and the amount of red decreasing proportionally to the hydrophilicity. The potentially charged residues are light blue. (The plots were made using the software created by Don Armstrong and Raphael Zidovetzki. Version: 0.10 p06 12/14/2001 DLA modified by Jim Hu.)

On the other hand, lactoferrampin belongs to Group 1 which consists of linear, cationic, and amphipathic-helical peptides. The alpha-helical amphipathic character of lactoferrampin has been compared with other Group I peptides like magainins, bombinins, cecropin A, and temporins and are depicted by the helical wheel representation of the peptides in which the charged and polar residues are found aligned along one side and most of the amino acids with nonpolar side chains occupy the opposite side of the helical cylinder (Figure 3). The spatial segregation of the hydrophobic and hydrophilic residues designates the amphipathic nature of the peptides [127, 128]. These peptides upon interaction with target membranes fold into an amphipathic *α*-helix with one face of the helix predominantly containing the hydrophobic amino acids and the opposite face the charged amino acids [129]. The presence of a prominent hydrophobic face is observed in the helical wheel representations of magainin 2, bombinin, and temporin (Figures 3(a), 3(b), and 3(c)) whereas a pronounced cationic domain is present on the hydrophilic surface of the helical wheel diagram of bovine lactoferrampin like that in cecropin A (Figures 3(d) and 3(e)). The positively charged domain is more distinct in bovine than human lactoferrampin (data not shown). The analysis suggests that there is very little similarity in the amino acid sequence within the group; however there is a distinct trend in the distribution of different types of residue, that is, hydrophobic and charged, polar, and so forth, within the secondary structure of the helix.

Lactoferricin has been shown to display a similarity with an antimicrobial peptide, magainin. Both peptides are able to traverse the bacterial cytoplasmic membrane [130]. Sequence similarities between lactoferricin and dermaseptin and magainins suggest that lactoferricin may act as an amphipathic alpha-helix [131]. The active hexapeptide fragment within bovine lactoferricin peptide showed distinct similarities with LF1-11 and other amphipathic tryptophan and arginine-rich antimicrobial peptides found in other sources apart from lactoferrin (Table 5). Bovine lactoferricin contains two tryptophan residues at positions 6 and 8 and two arginines at positions 4 and 5. These two amino acids have chemical properties which are one of the critical components of antimicrobial peptides [132].

## 7. Conclusion

Enzymatic digestion of lactoferrin results in the generation of antimicrobial peptides which display antimicrobial properties, in some cases, with greater potency than the native lactoferrin, possibly for the protection of neonates against the invading pathogens. These peptides, all from the N-lobe of lactoferrin, show a remarkable similarity to cationic antimicrobial peptides found in other invertebrate and vertebrate species. These peptides are conserved in lactoferrin, structurally and functionally in most species. Though there may be minor variations in the sequence and the conformational features among these lactoferrin peptides from various species, the basic framework tends to be similar and conserved. This indicates that these peptides play a significant role in the antimicrobial function of this protein. The antimicrobial effect of cationic peptides of different origins is due to cytoplasmic membrane disruption of the target cell as well as immunomodulation. The difference in their functional properties is due to the difference in their amino acid composition inspite of sharing amphipathic and cationic characteristics. The presence of all these antimicrobial peptides in a single domain of lactoferrin suggests that the protein acts on the membrane interface and disturbs the membrane integrity resulting in its antimicrobial activity. 

Since lactoferrin is found in the milk and is ingested throughout the life of all neonates and most adults, it may be an excellent agent for administration to humans. In the future, these lactoferrin peptides could serve as leads for drug development for antimicrobial therapy.

## Figures and Tables

**Table 1:** Amino acid sequences of LF1-11 from lactoferrin from six species.

	LF1-11	Lactoferrampin	Lactoferricin
Sequence	GRRRSVQWCAV	WNLLRQAQEKFGKDKSP	KCFQWQRNMRKVRGPPVSCIKRDS
pI	11.70	9.70	10.95
Secondary structure in the intact lactoferrin [X-ray crystallography]	Loop followed by *β*-strand	Amphipathic helix with a C-terminal tail	N-terminal amphipathic helix connected to a *β*-strand with a loop. The structural assembly is held together with a disulphide bond.
Secondary structure when isolated [NMR]	Not known	N-terminal amphipathic alpha-helical conformation across the first 11 residues and random C-terminus	N-terminal amphipathic helix and a random coil

**Table 2:** Comparison of amino acid sequences of LF1-11 from lactoferrin from six species.

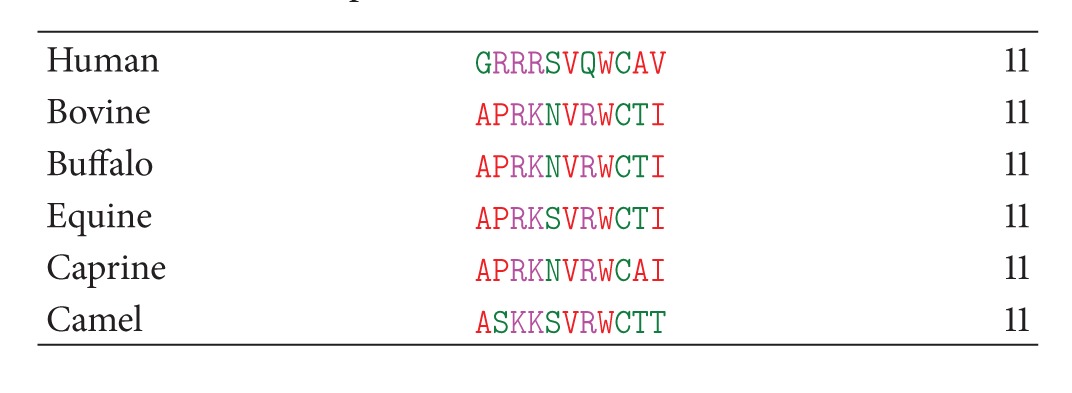

**Table 3:** Comparison of amino acid sequences of lactoferrampin from lactoferrin from six species.

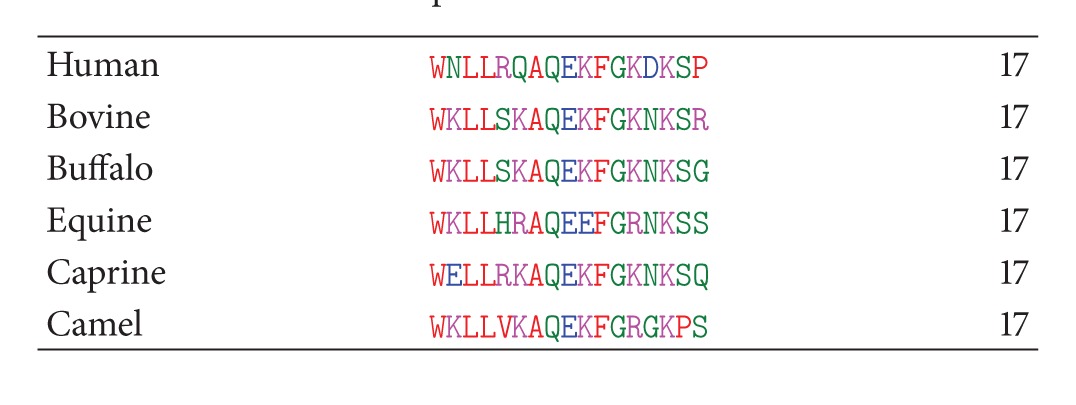

**Table 4:** Amino acid sequences of lactoferricin from lactoferrin from six species.

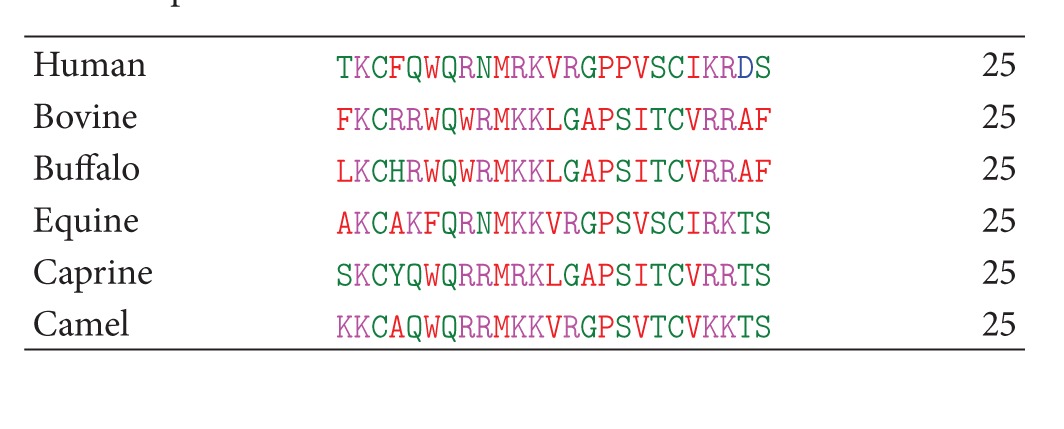

**Table 5:** Comparison of amino acid sequences of lactoferricin and other tryptophan and arginine containing antimicrobial peptides. The active hexapeptide of lactoferricin and its corresponding matching residues in other antimicrobial peptides are indicated in red.